# Increased Heat Pain Tolerance but Hyperalgesia to Tonic Inflammatory Pain in the CRND8 Mouse Model of Alzheimer’s Disease

**DOI:** 10.3233/JAD-230148

**Published:** 2023-10-24

**Authors:** Sara Merlo, Lara Costa, Santina Chiechio, Carla Letizia Busceti, Lucia Ciranna, Rosa Santangelo, Maria Angela Sortino, Francesco Fornai, Ferdinando Nicoletti, Agata Copani

**Affiliations:** a Department of Biomedical and Biotechnological Sciences, University of Catania, Catania, Italy; b Department of Clinical and Experimental Medicine, University of Messina, Messina, Italy; c Department of Drug and Health Sciences, University of Catania, Catania, Italy; d Oasi Research Institute - IRCCS, Troina, Italy; e Department of Molecular Pathology, IRCCS Neuromed, Pozzilli, Italy; f Department of Translational Research and New Technologies in Medicine and Surgery, University of Pisa, Pisa, Italy; g Department of Physiology and Pharmacology, University Sapienza, Rome, Italy; h Institute of Crystallography, National Council of Research, Catania Unit, Catania, Italy

**Keywords:** Alzheimer’s disease, dementia, dorsal root ganglia, nociceptive neurons, spinal cord, transgenic AD mice

## Abstract

**Background:**

The effects of Alzheimer’s disease (AD) pathology on the experience of pain are poorly understood.

**Objective:**

To understand the pathophysiological mechanisms underlying pain sensory transmission in the transgenic mouse model of AD, CRND8.

**Methods:**

We explored AD-related pathology in the spinal cord and dorsal root ganglia of 18-week-old female CRND8 mice. We assessed nociceptive responses to both acute heat stimuli and persistent inflammatory pain in CRND8 mice and non-transgenic (non-Tg) littermates. In addition, we searched for differences in biochemical correlates of inflammatory pain between CRND8 and non-Tg mice. Finally, we investigated the excitability of dorsal horn noc iceptive neurons in spinal cord slices from CRND8 and non-Tg mice.

**Results:**

We demonstrated the presence of intracellular AD-like pathology in the spinal cord and in the dorsal root ganglia nociceptive sensory neurons of CRND8 mice. We found that CRND8 mice had a reduced susceptibility to acute noxious heat stimuli and an increased sensitivity to tonic inflammatory pain. Tonic inflammatory pain correlated with a lack of induction of pro-opiomelanocortin in the spinal cord of CRND8 mice as compared to non-Tg mice. Electrophysiological recording in acute spinal cord slice preparations indicated an increased probability of glutamate release at the membrane of dorsal horn nociceptive neurons in CRND8 mice.

**Conclusion:**

This study suggests that an increased thermal tolerance and a facilitation of nociception by peripheral inflammation can coexist in AD.

## INTRODUCTION

Several lines of evidence, including self-report, observational pain scales, or nurse ratings, have indicated a decrease in pain prevalence and severity in Alzheimer’s disease (AD) patients [1, 2]. As such, historical data have shown that AD patients receive fewer analgesics than their age-matched controls [3]. However, more recent population-based studies report that patients with AD and related dementia are equally or more likely than cognitively unimpaired individuals to receive analgesics for pain management [4, 5], suggesting a greater perceived need of pain medications with the risk of inappropriate prescription practices. Actually, because pain is an individual experience and its assessment may be biased by the presence of a cognitive deficit, the entity of pain experienced by AD subjects remains largely elusive.

AD neuropathology may affect central pain processing, which relies on spinal circuits projecting to the supraspinal medial and lateral pain systems. The former plays a crucial role in the emotional and cognitive features of pain, and in the autonomic responses evoked by pain; the latter is responsible for the sensory/discriminative features of noxa. Because the neuropathology affects the medial pain system and spares the lateral pain system [6], the general belief has been that AD reduces the emotional and cognitive response to painful stimuli [2]. However, functional MRI of brain response to noxious mechanical pressure has shown that activity in both medial and lateral pain pathways is preserved in AD patients who, nonetheless, have an increased threshold for pain sensitivity [7].

Few studies have addressed the nociceptive behavior of transgenic animal models of AD. Both CRND8 mice, harboring two familial AD-related mutations of amyloid precursor protein (AβPP), and TASTPM mice, bearing AβPP and presenilin-1 mutant transgenes, exhibit a reduced sensitivity to acute noxious heat stimuli. Curiously, TASTPM mice respond normally to peripheral inflammatory pain [8], whereas CRND8 mice show a reduced sensitivity to it [9]. Unlike CRND8 and TASTPM mice, 3xTg-AD mice, bearing AβPP, presenilin-1, and tau mutant transgenes, show a preserved response to thermal pain from asymptomatic to advanced stages of the disease [10].

The aim of this study was to use CRND8 mice for a better understanding of the pathophysiological mechanisms underlying pain sensory transmission. Specifically, we used 18-week-old female CRND8 mice. At this age, CRND8 mice display diffuse amyloid-β (Aβ) pathology accompanied by cognitive deficits [11], with females expected to be more sensitive than male to painful stimuli [12]. We explored AD-related pathology in the spinal cord and dorsal root ganglia (DRG) of CRND8 mice and assessed their behavioral responses to both acute thermal pain and persistent inflammatory pain as compared to non-Tg littermates. Biochemical correlates of inflammatory pain were compared in CRND8 and non-Tg mice. Finally, we searched for indications about the excitability of lamina II dorsal horn (DH) neurons from spinal cord slice preparations of CRND8 and non-Tg mice.

## MATERIALS AND METHODS

### Transgenic mouse model: CRND8

Heterozygous transgenic CRND8 male mice on a 129/SVE genetic background were originally obtained from the Centre for Research in Neurodegenerative Diseases, University of Toronto, Ontario, Canada. The colony has been established in the Animal House of the University of Catania, Italy. CRND8 mice express the human AβPP695 with the Swedish mutation (KM670/671NL) and the Indiana mutation (V717F) under the control of the hamster prion (PrP) gene promoter [11]. F1 transgenic female CRND8 and non-transgenic female littermates (non-Tg) were used for the experiment. Animal care and experimentation were implemented according to institutional guidelines with approval of the Italian Ministry of Health (authorization number 205/2016-PR).

### Immunohistochemistry

Brain, spinal cord, and DRG tissue samples from CRND8 transgenic mice or their non-Tg littermates were fixed overnight at 4°C in Carnoy’s solution (ethyl alcohol (60%), acetic acid (10%), and chloroform (30%)). Afterwards, all samples were paraffin embedded and then cut to obtain 10μm sections.

For immunohistochemical staining, sections were deparaffinized with xylene and rehydrated by serial dilutions of ethanol. For antigen retrieval, sections were treated with 70% formic acid at room temperature (RT) for 30 min. After that, sections were treated at RT for 15 min with 0.1% triton X-100 for permeabilization and then for 10 min with 3% hydrogen peroxide to block endogenous peroxidase activity. Afterwards, sections were treated at RT for 1 h with 10% normal horse serum for blocking non-specific binding and then incubated overnight at 4°C with the mouse primary antibody anti-β-amyloid, 1–16 (clone 6E10) (1:100, code 803001, Biolegend, San Diego, CA) or, for 48 h at 4°C, with the rabbit primary antibody anti-β-amyloid 1–40 (1:20, code AB5737, Millipore, Burlington, MA). Finally, sections were incubated for 10 min at RT with a secondary biotinylated anti-mouse IgG made in horse (1:200, code BA-2000, Vector Laboratories, Burlingame, CA) or for 2 h at RT with a secondary biotinylated anti-rabbit IgG made in horse (1:200, code BA-1000, Vector Laboratories, Burlingame, CA) and then for 5 min (following the secondary biotinylated anti-mouse IgG) or 2 h (following the secondary biotinylated anti-rabbit IgG) at RT with horseradish peroxidase streptavidin (1:100, code SA-5004-1, Vector Laboratories). Either 3,3’-diaminobenzidine (DAB) or DAB/nickel substrate working solutions were used.

For double fluorescence analysis, additional sections subjected to deparaffinization, antigen retrieval, permeabilization and blocking of non-specific binding sites (with a mixture containing 10% normal horse serum and 10% normal donkey serum) were incubated overnight at 4°C with a mixture containing the following primary antibodies: mouse monoclonal anti-β-amyloid, 1-16 (clone 6E10) (1:100, code 803001, Biolegend), and rabbit polyclonal anti-TrkA (1:100, code SAB1305370, Sigma-Merck) or rabbit polyclonal anti-P2X3 (1:100, code AB5895, Millipore, Burlington, MA). Afterwards, all slices were incubated for 60 min at RT with the following secondary antibodies: fluorescein horse anti-mouse IgG (1:100, code FI-2000, Vector Laboratories) and Cy3 conjugated donkey anti-rabbit IgG (1:300, code AP182 C, Life Technologies). For antigen retrieval, sections were treated with 70% formic acid at RT for 30 min. Co-labeling analyses of 6E10/anti-TrkA and 6E10/anti-P2X3 were carried out by Adobe Photoshop on merged fluorescence images of DRG sections from CRND8 mice. A total of 328 cells from 3 DRG sections were scored for the 6E10/anti-TrkA co-labeling, whereas a total of 130 cells from 2 DRG sections were scored for the 6E10/anti-P2X3 co-labeling. To determine cellular positivity, fluorescence intensity values in the red (anti-TrkA or anti-P2X3) and green (6E10) channels for each single cell present in a given DRG section were calculated after background subtraction.

### Behavioral experiments

Non-Tg and CRND8 female mice (18-week-old) were used to evaluate pain behavior. To minimize variability, tests were performed between 08:00 and 12:00 am. Acute thermal sensitivity was assessed by the tail flick-test and Hargreaves test, whereas inflammatory pain was evaluated by the formalin test.

Thermal sensitivity: for the tail-flick test, a noxious heat stimulus was applied via a focused, radiant heat light source (IITC) to the middle of the tail. The baseline tail-flick latency was calculated as the mean of three different measurements taken at 15 min intervals. For the Hargreaves test, thermal sensitivity was measured using a Hargreaves-type apparatus (IITC) [13]. Paw-withdrawal latency was calculated as the mean of three to five different measurements taken at 15-min intervals.

Formalin test: a formalin solution (5%; 10μl in saline; Sigma-Merck) was injected subcutaneously into the plantar surface of the right hind paw of mice. After the injection, mice were immediately placed in a behavioral box (20×15×15 cm) and nociceptive responses that include licking, lifting and shaking of the injected paw were observed and recorded for 1 h. Formalin scores were separated into two phases, phase I (0–10 min) and phase II (15–60 min). The mean behavioral score was monitored for 60 min and recorded in 5 min intervals for each of the two phases.

### Western blot analysis

The ipsilateral and contralateral lumbar tracts of the spinal cord were collected after the formalin test and lysed in RIPA buffer (Sigma-Merck) supplemented with anti-protease and anti-phosphatase cocktails (Sigma-Merck). Samples were sonicated and centrifuged at high speed to remove insoluble debris. Supernatants were collected and protein concentration was determined with the Bradford reagent (Sigma-Merck) using a Varioskan^TM^ plate reader for measurements. Fifty μg of each sample/lane were loaded on 4–15% gradient precast gels (Bio-rad, Hercules, CA, USA) and subjected to sodium dodecyl sulfate-poly-acrylamide gel electrophoresis (SDS-PAGE) followed by transfer on nitrocellulose membranes (Hybond ECL, Amersham Biosciences Europe GmbH, Milan, Italy) in a Transblot semi-dry transfer cell (Bio-rad) for 70 min. Membranes were blocked with Blocker FL Fluorescent Blocking buffer (Thermofisher) for 30 min and incubated on a rocking platform with primary antibodies for 24–48 h at 4 °C, followed by wash and incubation with appropriate fluorochrome-conjugated secondary antibodies for 1 h at RT. Primary antibodies: rabbit anti-Fos (1:1000; cat. no. ab190289, Abcam); rabbit anti-phospho-p44/42 MAPK (Erk1/2; 1:800, cat. no. 9101, Cell Signaling Technologies), mouse anti-Met/Leu Enkefalin (NOC1/35) (1:150; cat. no. sc-47705, S. Cruz Biotechnology), rabbit anti-pro-opiomelanocortin (POMC; 1:500, cat. no. E-AB-53251, Elabscience^®^), rabbit anti-β-actin (1:3000; cat. no. A2066, Merck), rabbit anti-p44/42 MAPK (Erk1/2; 1:1000, cat. no. 9102, Cell Signaling Technologies), mouse anti-Glyceraldehyde 3-phosphatedehydrogenase (6C5) (GAPDH; 1:5000, cat. no. MAB374, Merck). Secondary antibodies: IR800-conjugated anti-rabbit (1:1000; Li-cor) and Alexa-Fluor 488Plus-conjugated anti-mouse (1:5000; cat. no. A32723, Thermofisher). Bands were detected with the iBright FL1500 Imaging System (Thermofisher). Band intensity was analyzed using the ImageJ software.

### Electrophysiology

Spinal cord slices were prepared from non-Tg and CRND8 mice using a standard protocol [14]. For this set of experiments, 11-week-old mice were used as, at this age, it was easier to obtain intact slices and a remarkable Aβ production is known to be present [11]. Following decapitation, the spinal cord was rapidly removed by hydraulic extrusion. The lumbar part was transferred to ice-cold (0°C) calcium-free sucrose-based artificial cerebrospinal fluid (composition in mM: sucrose 252, NaH_2_PO_4_ 1.25, KCl 2.5, NaHCO_3_ 26, glucose 10, MgCl_2_ 2; pH 7.3, continuously bubbled with 95% O_2_/5% CO_2_,) and embedded in low gelling temperature agarose (4%, Sigma-Merck). Transverse slices (300μm) were cut with a vibratome (VT1200, Leica) and were transferred to a storage chamber containing artificial cerebrospinal fluid (ACSF; composition in mM: NaCl 126, NaH_2_PO_4_ 1.25, KCl 2.5, CaCl_2_ 2, MgCl_2_ 2, glucose 10, NaHCO_3_ 26; pH 7.3) continuously bubbled with 95% O_2_/5% CO_2_. After a recovery period of 1 h at room temperature (20°C), a slice was transferred to the recording chamber of a patch clamp set up and visualized using two water-immersion objectives (HCX/APO L 10X/0.30 and 40X/0.80) with infrared differential interference contrast (DMLFS microscope, Leica, Wetzlar, Germany) connected with an infrared-sensitive camera. Spontaneous and miniature excitatory post-synaptic currents (sEPSCs and mEPSCs) were recorded under whole-cell voltage clamp from visually identified lamina II DH neurons (holding potential –70 mV). EPSC traces were acquired with an EPC7-plus amplifier (HEKA, Germany) and a 1401 Plus interface (Cambridge Electronic Design, CED. U.K.), filtered at 5 kHz and digitized at a 10 kHz sampling rate using the software Signal (CED, U.K.). The recording micropipette (resistance 1.5–3 MΩ) was filled with intracellular solution (in mM: K-gluconate 140; HEPES 10; NaCl 10; MgCl_2_ 2; EGTA 0.2; Mg-ATP 3.5; Na-GTP 1; pH 7.3). AMPA receptor-mediated sEPSCs were recorded in ACSF (flow rate 1.5 ml/min) containing (-)-bicuculline methiodide (5μM, Sigma-Merck) and D-(-)-2-Amino-5-phosphonopentanoic acid (D-AP5, 50μM, Tocris) and strychnine (10μM, Sigma-Merck). After 5 min of sEPSC recording, mEPSCs were recorded from the same neurons in ACSF containing bicuculline (5μM); D-AP5 (50μM), strychnine (10μM), and tetrodotoxin (TTX, 0,1μM, HelloBio). Data analysis was performed using the Clampfit module of PClamp 10 software (Molecular Devices, Sunnyvale, CA, USA) to measure sEPSC and mEPSC frequency (Hz, n of events/sec), peak amplitude (pA), rise time (ms; time to reach from 10 to 90% of peak amplitude) and decay time (ms; time to decay from 90 to 10% of peak amplitude).

### Statistical analysis

Quantitative data were expressed as the mean±standard error (SEM). A two-way or a two-way repeated measures ANOVA, with Bonferroni post-test for all pairwise multiple comparisons, was carried out to estimate data changes according to different categorical variables (i.e., genotype and injection side or genotype and post-injection time phase). Moreover, where indicated, *p* values were calculated using unpaired *t* test, either one-tailed or two-tailed as appropriate. Welch’s correction was applied in the case of unequal sample sizes. Analysis was carried out using either SigmaPlot 12.5 or GraphPad Prism 6.

## RESULTS

### AβPP/Aβ was expressed in DRG nociceptive sensory neurons

**Fig. 1:**
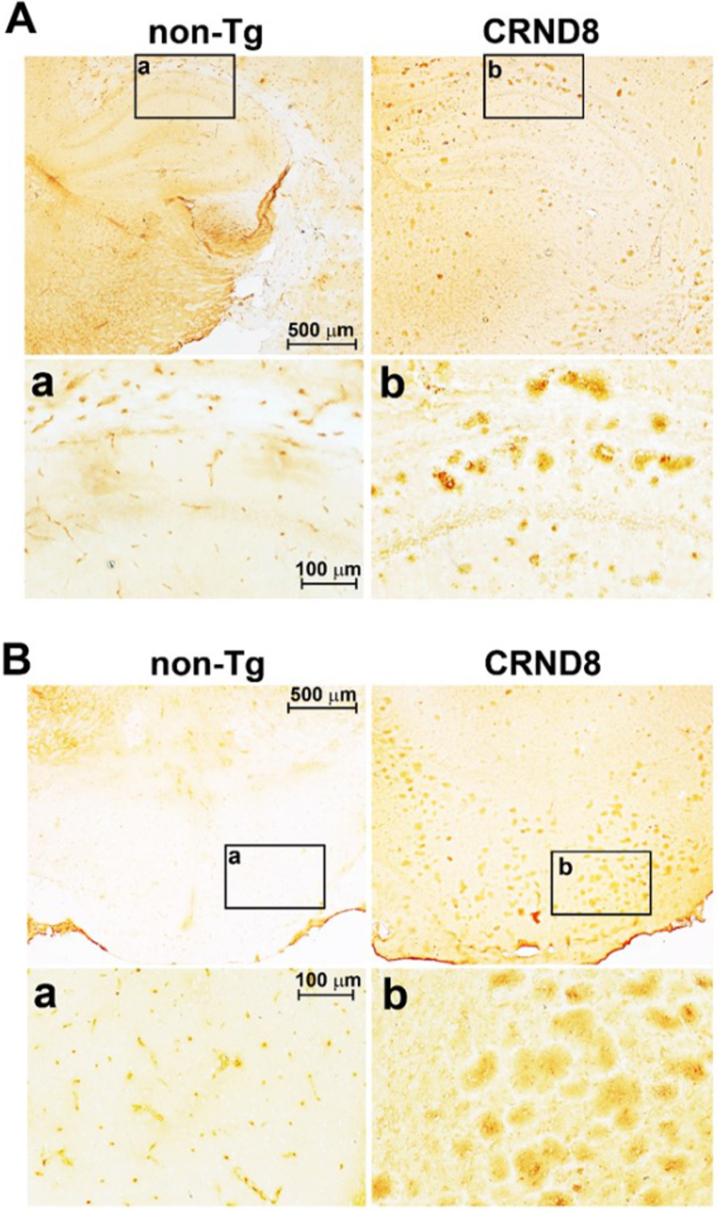
Aβ plaques were clearly visible in the brain of CRND8 mice. Representative histological sections of CRND8 and non-Tg brains stained with 6E10 antibody. In A, Aβ plaques were visible throughout the brain of CRND8 but not non-Tg mice, with a) and b) referring to zoomed-in insets of the hippocampus in non-Tg and CRND8 mice, respectively. In B, brain slices containing the hypothalamus were imaged at low magnification, with a) and b) referring to zoomed-in insets of the hypothalamus in non-Tg and CRND8 mice, respectively.

**Fig. 2:**
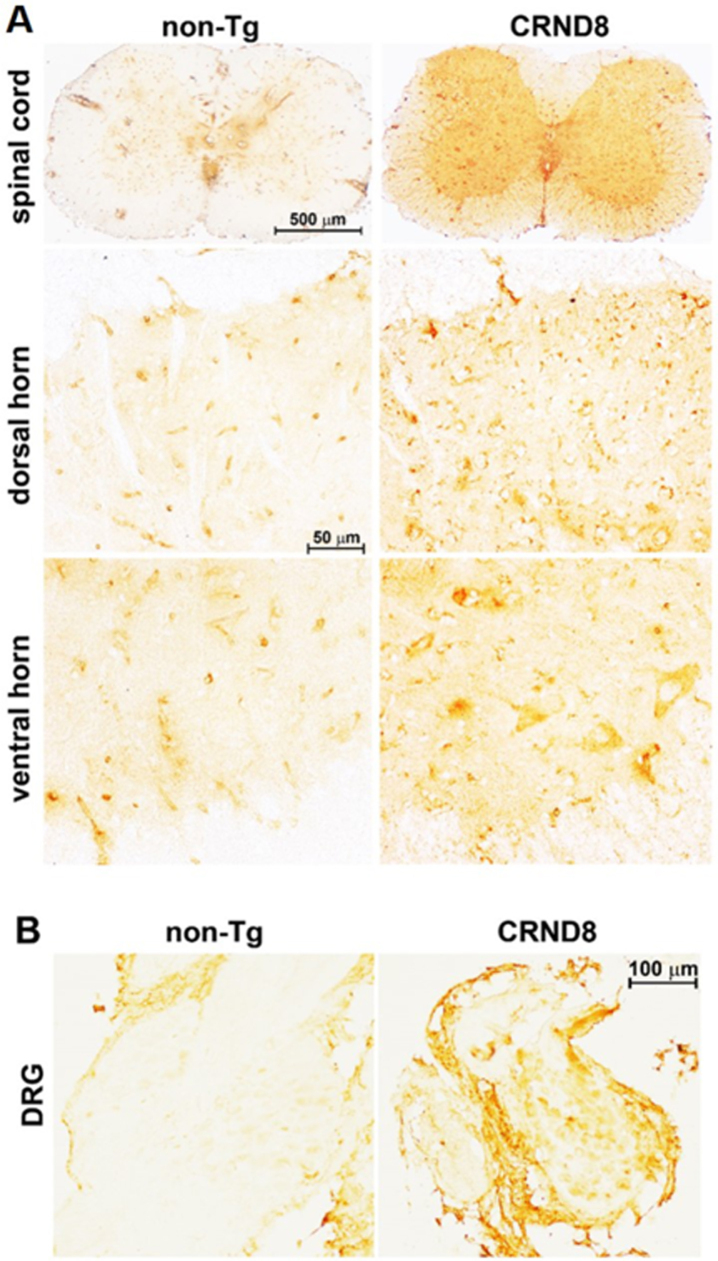
Cellular AβPP/Aβ was visible in the spinal cord and in the DRG of CRND8 mice. Representative AβPP/Aβ immunostaining of CRND8 and non-Tg lumbar spinal cord (A) and DRG (B) sections. AβPP/Aβ immunostaining, by 6E10 antibody, profiled cell bodies in the dorsal horns, in the ventral horns and in the DRG of CRND8 mice. Aβ plaques were not found.

CRND8 mice exhibit cognitive deficits and Aβ deposition by the age of 3 months [11]. As expected, we observed amyloid plaques in the brain of 18-week-old female CRND8 mice (Fig. 1A, B - insets b); however, no plaque pathology was found in the spinal cord (Fig. 2A, right side). By using 6E10, a monoclonal antibody that recognizes both human AβPP and Aβ, a cellular immunostaining of AβPP/Aβ was observed in the spinal cord (in both the dorsal and ventral horns) (Fig. 2A, right side) and in the DRG of CRND8 mice (Fig. 2B, right side), but not non-Tg mice (Fig. 2A, B, left side). About 80% of DRG neurons stained for AβPP/Aβ (78 ± 6.565 %, *n* = 5, with n representing the number of analyzed DRG sections).

**Fig. 3:**
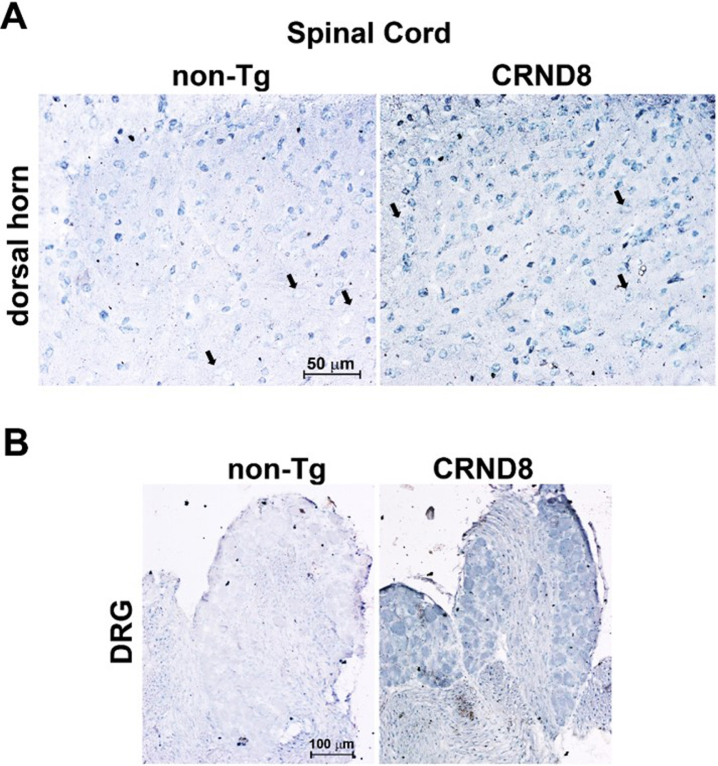
Cellular Aβ_40_ was visible in the lumbar dorsal horns and in the DRG of CRND8 mice. Representative Aβ_40_ immunostaining of cell bodies in CRND8 and non-Tg lumbar dorsal horn sections (A). Black arrows indicate large cell bodies that were immunolabeled in CRND8 but not in non-Tg mice (A). In B, representative photomicroscopies of CRND8 and non-Tg DRG sections immunostained for Aβ_40_. Aβ_40_ immunostaining was present in CRND8 but not in non-Tg mice.

An additional antibody, which recognizes the free C-terminal end of human Aβ_40_, immunostained the DH of both CRND8 and non-Tg mice, likely because of the cross-reactivity between human and mouse Aβ. Noteworthy, CRND8 mice exhibited a greater number of immunolabeled cells in the DH, including large neuronal bodies that were spared in non-Tg mice (Fig. 3A). DRG neurons from CRND8 mice were clearly immunostained for Aβ_40_ unlike those from non-Tg mice (Fig. 3B). However, while DRG neurons from non-Tg mice never stained for Aβ_40_, probably due to a low endogenous level of the peptide, the extent of cellular immunolabeling for Aβ_40_ was variable among the ganglia of the same CRND8 animal (not shown). Overall, Aβ_40_ cellular immunolabeling confirmed that human AβPP-derived Aβ peptides were being detected in the DRG of CRND8 animals.

**Fig. 4:**
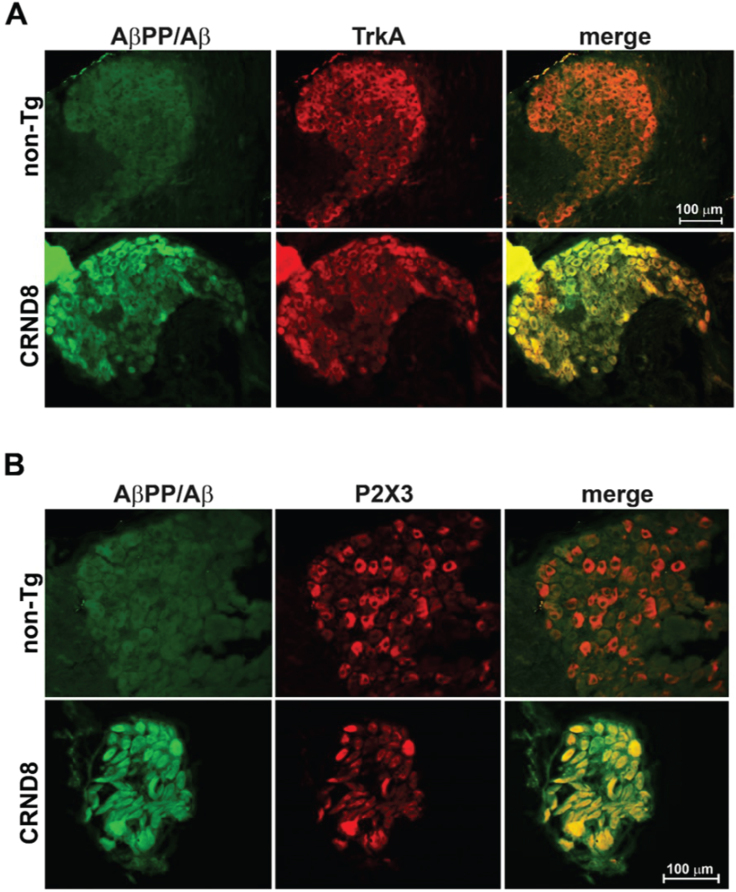
Sensory nociceptive neurons in the DRG of CRND8 mice all expressed AβPP/Aβ. Representative fluorescence images of DRG neurons of CRND8 mice labeled with AβPP/Aβ and either TrkA (A –bottom panel) or P2X3 (B –bottom panel) as markers for peptidergic and non-peptidergic nociceptors, respectively. Representative fluorescence images of DRG neurons of non-Tg mice labeled with AβPP/Aβ and either TrkA (A –top panel) or P2X3 (B –top panel) are shown for comparison.

In the DRG of CRND8 mice, virtually all nociceptive neurons, labeled with either TrkA or P2X3, markers for nociceptive neurons of the peptidergic [15] and non-peptidergic type [16], respectively, were immunoreactive for AβPP/Aβ (TrkA^+^/AβPP/Aβ^+^: 91.667 ± 5.239 %, *n* = 3; P2X3^+^/AβPP/Aβ^+^: 100%, *n* = 2, with n representing the number of analyzed DRG sections) (Fig. 4A, B –bottom panels), whereas none co-labeled with AβPP/Aβ in non-Tg mice (Fig. 4A, B –top panels).

### Pain response to acute heat stimuli was reduced in CRND8 mice

**Fig. 5:**
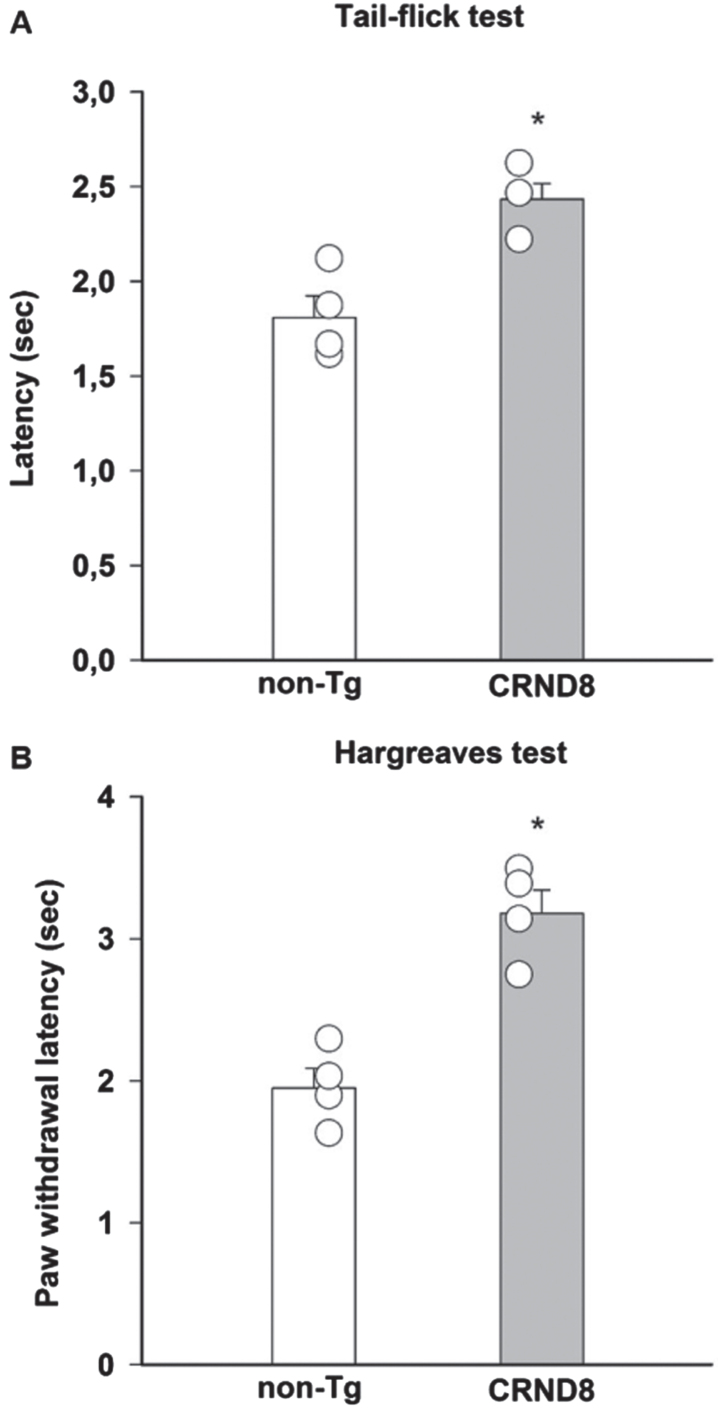
Reduced pain responses to noxious thermal stimulation in CRND8 mice. A significant increase of pain latency, induced by radiant heat light source and measured by tail-flick latency (A) and paw withdraw latency (Hargreaves, B), was observed in CRND8 mice compared to non-Tg mice (*p < 0.05, two-tailed *t* test). Data are represented as mean±SEM in bar graphs showing individual values (*n* = 4 per genotype).

Because of the putative expression of AβPP/Aβ in nociceptive neurons, including the peptidergic subtype that is necessary for noxious heat activation of DH neurons [17], we initially evaluated the susceptibility of CRND8 mice to harmful heat stimuli. CRND8 mice exhibited increased tail flick (Fig. 5A) and paw withdrawal latencies (Fig. 5B) to thermal stimulation compared with non-Tg mice, which were indicative of a compromised response to heat stimuli [18]. Locomotor function was comparable in CRND8 and non-Tg mice (not shown).

### Tonic but not acute inflammatory pain was increased in CRND8 mice

**Fig. 6:**
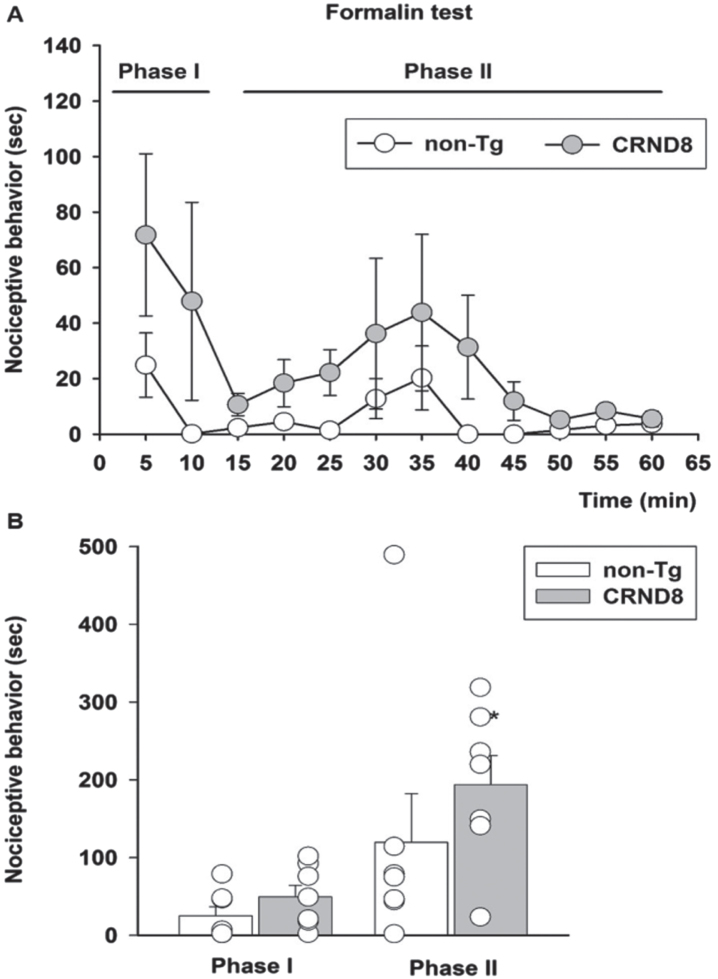
Enhanced pain behavior after formalin injection in CRND8 mice. In (A) the time course of formalin-induced pain behavior (as duration of licking and flinching responses) in CRND8 and non-Tg mice is presented. In (B), pain responses were classified as phase I (0–10 min) and phase II (15–60 min) and were represented as mean±SEM in bar graphs showing individual values (*n* = 7 per genotype). A two-way repeated measures ANOVA with Bonferroni post-test for all pairwise multiple comparisons was carried out to analyze the effects of genotype and phase on pain responses. There was a statistically significant difference in pain response between CRND8 and non-Tg mice within phase II (*p* = 0.015).

As a model of persistent pain, more closely resembling clinical pain, and involving the activation of P2X3^+^ nociceptors [19], we used the formalin test [20]. Following formalin injection, both non-Tg and CRND8 mice displayed the typical biphasic curve of licking behavior of the injected paw (Fig. 6A). The first (early) phase of the formalin test, starting soon after formalin injection and lasting for about 10 min, reflects the acute inflammatory pain triggered by the direct activation of peripheral nociceptors. The second (late) phase of the test, occurring approximately 15-60 min after formalin injection, reflects the persistence of nociception due to the sensitization of DH neurons to the noxious stimulus [21]. Pain behavior was not significantly different between CRND8 and non-Tg mice during the first phase of the test; however, as compared to non-Tg mice, CRND8 mice showed a significant increase of nociceptive behavior during the second phase of the test (Fig. 6B). Hence, CRND8 mice exhibited an increased threshold to harmful heat stimuli (Fig. 5) but, curiously, a facilitation of nociception by peripheral tissue injury (Fig. 6).

### Lack of correlation between tonic inflammatory pain and the expression of p-ERK and Fos in the spinal cord of formalin-injected CRND8 mice

**Fig. 7:**
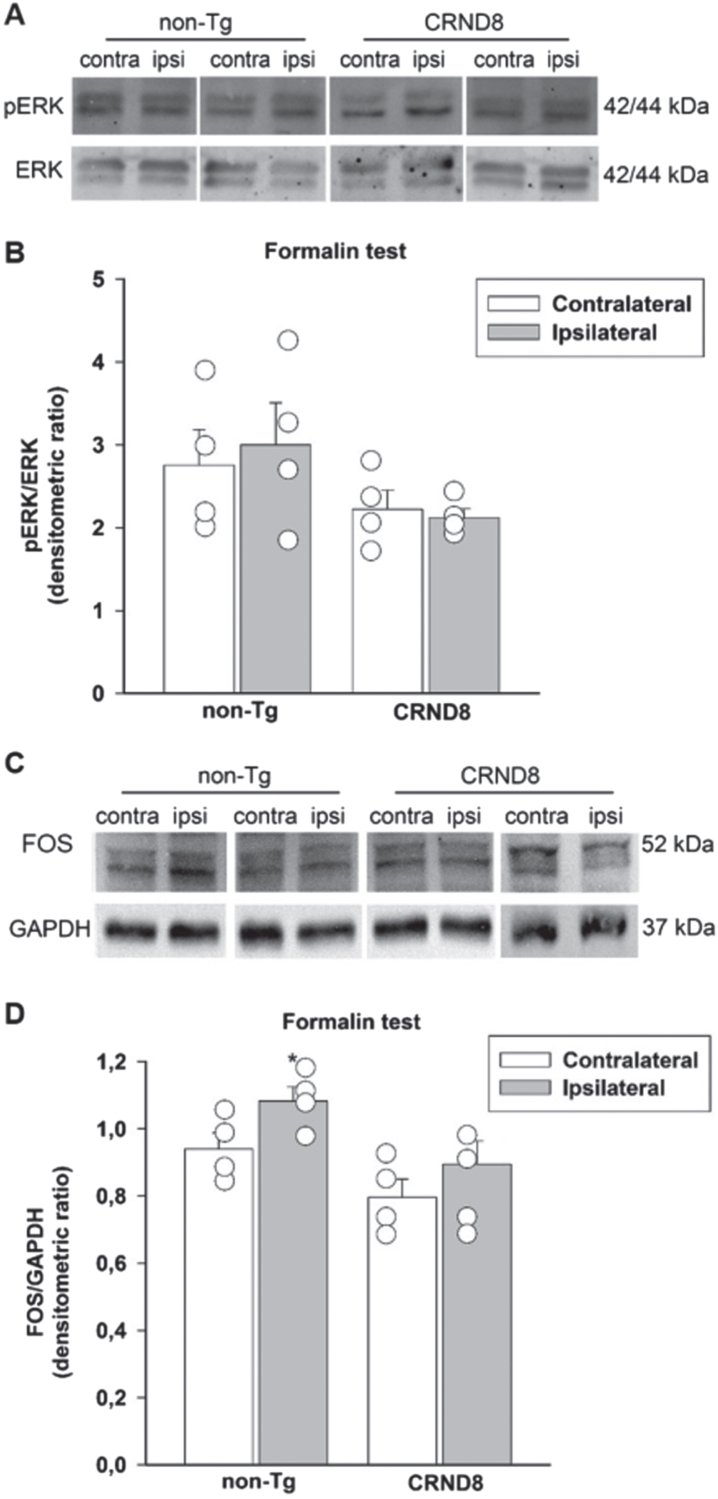
Formalin injection did not induce the expression of Fos and p-ERK in the CRND8 mice spinal cords. Representative western blot images of p-ERK (A) and Fos (C) expression in lumbar specimens 60 min after formalin injection. Two animals per genotype, and the corresponding protein samples obtained from the contralateral (contra) and ipsilateral (ipsi) sites to the formalin injection, are shown. The total ERK bands and the GAPDH band are shown as loading controls for A and C, respectively. Densitometric values of p-ERK (B) and Fos (D) bands from four different animals per genotype, normalized against ERK or GAPDH, are presented in bar graphs showing individual values. A two-way ANOVA with Bonferroni post-test for all pairwise multiple comparisons was carried out to analyze the effects of genotype and side on either p-ERK or Fos expression. There was a statistically significant difference in Fos expression between the ipsilateral and the contralateral side in non-Tg mice (*p* = 0.003). No other significant difference was found.

Both the phosphorylated signaling kinase, ERK, (p-ERK) and the nuclear protein Fos are transiently expressed by neurons in response to nociceptive stimulation and their expression eventually persists during the late phase of formalin test [22]. We examined the expression of p-ERK and Fos in the lumbar segment of the mice spinal cords, on both the ipsilateral and contralateral side to the formalin injection site. Sixty min after formalin injection, spinal cord specimens were obtained from CRND8 and non-Tg mice and processed for western blot analysis. Both CRND8 and non-Tg mice failed to show an increased expression of p-ERK (Fig. 7A, B) in the ipsilateral side to the formalin injection, whereas only non-Tg mice exhibited an increased expression of Fos (Fig. 7 C, D) ipsilaterally to the formalin injection. This evidence suggested that the peak expression of p-ERK was rapid and transient in CRND8 mice as well as in non-Tg mice, whereas the expression of Fos increased over a different time frame in CRND8 and non-Tg mice.

### Tonic inflammatory pain was not related to a reduced expression of enkephalins in the spinal cord of formalin-injected mice

**Fig. 8:**
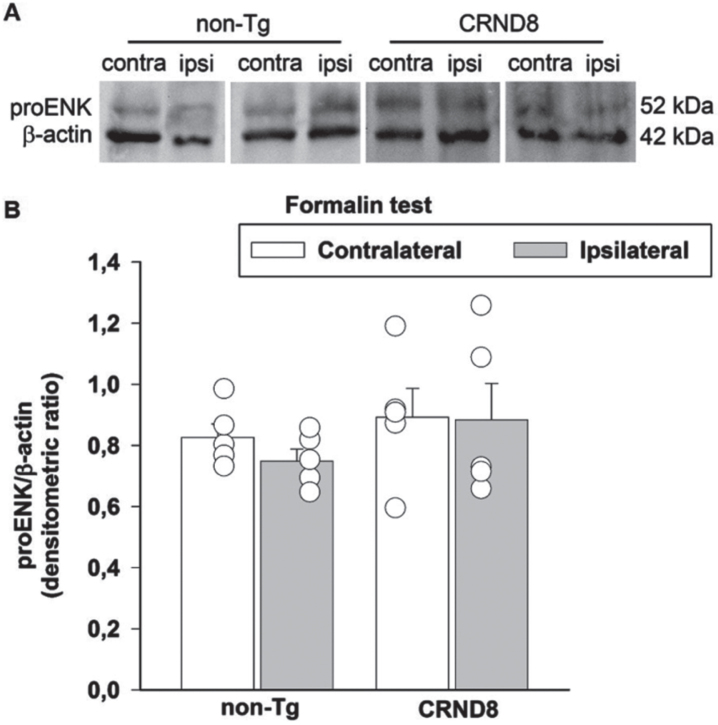
Formalin injection did not reduce the expression of proenkephalin in the mice spinal cords. Representative western blot image of proenkephalin (proENK) expression in lumbar specimens 60 min after formalin injection (A). Two animals per genotype, and the corresponding protein samples obtained from the contralateral (contra) and ipsilateral (ipsi) sites to the formalin injection, are shown. The β-actin band is shown as loading control. Densitometric values of proENK bands from five different animals per genotype, normalized against β-actin, are presented in bar graphs showing individual values (B). Values represent the mean±SEM. A two-way ANOVA with Bonferroni post-test for all pairwise multiple comparisons was carried out to analyze the effects of genotype and side on proENK expression. No significance was found.

Formalin can induce a transient activation of enkephalinergic neurons within spinal zones receiving the nociceptive inputs [23]. Therefore, we assessed the possible correlation between the levels of the endogenous opioid peptides, Leu/Met enkephalins and the nocifensive behavior of formalin-injected mice. We examined the expression of proenkephalin, the opioid polypeptide hormone from which Leu/Met enkephalins are derived, in the lumbar segment of the mice spinal cords, on both the ipsilateral and contralateral side to the formalin injection site. As in the case of Fos analysis, 60 min after formalin injection, spinal cord specimens were obtained from CRND8 and non-Tg mice and processed for western blot analysis. The antibody we used is able to detect proenkephalin and the processed peptides Leu/Met enkephalins. Expression signals of proenkephalin were not modified by formalin injection in either CRND8 or non-Tg mice, which exhibited not dissimilar proenkephalin signals on the contralateral side to the injection (Fig. 8A, B). Expression signals for Leu/Met enkephalins were never observed (not shown). This evidence suggested that the endogenous spinal tone of enkephalins was not related to the late phase of formalin-induced nocifension in CRND8 mice as well as in non-Tg mice.

### Tonic inflammatory pain correlated with a lack of induction of POMC/β-endorphin in the spinal cord of CRND8 mice

**Fig. 9:**
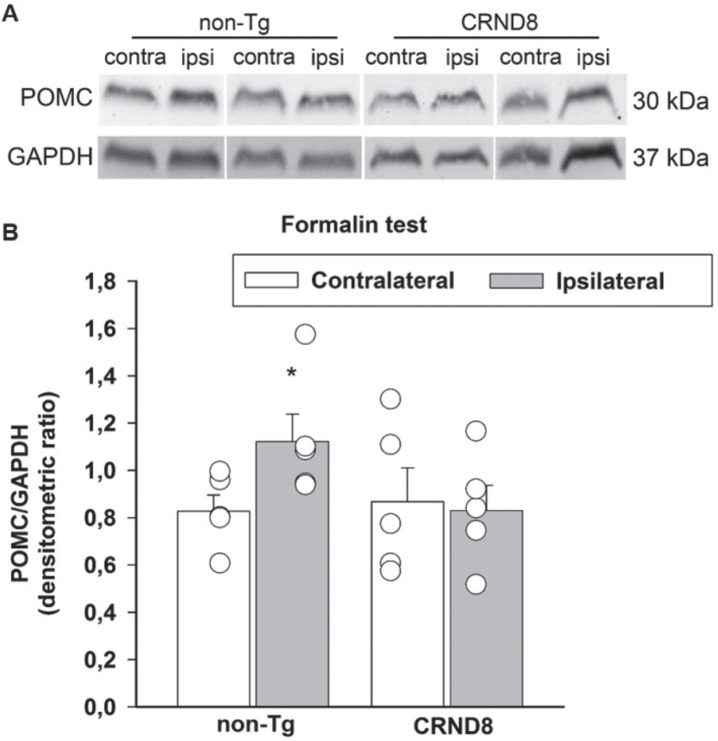
Formalin injection induced the expression of POMC in the spinal cord of non-Tg mice but not CRND8 mice. Representative western blot image of pro-opiomelanocortin (POMC) expression in lumbar specimens 60 min after formalin injection (A). Two animals per genotype, and the corresponding protein samples obtained from the contralateral (contra) and ipsilateral (ipsi) sites to the formalin injection, are shown. The GAPDH band is shown as loading control. Densitometric values of POMC bands from five different animals per genotype, normalized against GAPDH, are presented in bar graphs showing individual values (B). Values represent the mean±SEM. A two-way ANOVA with Bonferroni post-test for all pairwise multiple comparisons was carried out to analyze the effects of genotype and side on POMC expression. There was a statistically significant difference in POMC expression between the ipsilateral and the contralateral side in non-Tg mice (*p* = 0.006). No other significant difference was found.

Formalin has been found to increase the endogenous opioid peptide, β-endorphin, both in the hypothalamus and in the spinal cord [24]. β-endorphin is involved in spinal pain processing trough descending projections to, and intrinsic circuits of, the spinal cord, where pro-opiomelanocortin (POMC), the precursor of β-endorphin, is found [25]. Hence, we hypothesized that a failure to increase POMC following formalin injection could account for the hyperalgesia that CRND8 mice exhibited in the late phase of the formalin test. Western blot analysis of lumbar spinal cord specimens showed that the expression signals of POMC were not significantly different in CRND8 and non-Tg mice in the contralateral side to formalin injection. Interestingly, in CRND8 mice, POMC expression signals did not increase in the ipsilateral side to formalin injection, as they did in non-Tg mice (Fig. 9A, B). This evidence suggested that a reduced tone of POMC/β-endorphin could contribute to the late phase of formalin-induced nocifension in CRND8 mice.

### Enhanced probability of glutamate release at the membrane of nociceptive neurons in CRND8 mice

The evidence above supported the idea that CRND8 mice may have an impaired modulation of pain response or perception; yet, any information on the excitability of DH nociceptive neurons *per se* was missing. Primary afferent fibers transmit impulses from the periphery to nociceptive neurons that are mainly found in the superficial DH (laminae I-II). Glutamate released by primary afferents activate AMPA receptors, which set the baseline response of DH neurons to noxious stimuli [26]. Therefore, we decided to investigate whether the baseline activity of DH nociceptive neurons was increased in CRND8 mice by measuring AMPA receptor-mediated synaptic currents.

**Fig. 10:**
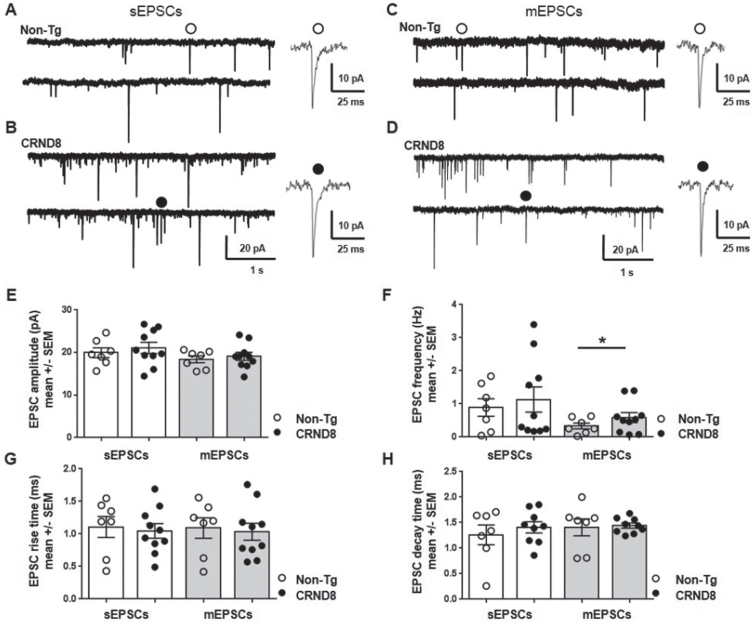
Pre-synaptic enhancement of AMPA receptor-mediated synaptic currents in lamina II DH neurons from CRND8 mice. AMPA receptor-mediated sEPSCs were recorded under whole cell voltage clamp from non-Tg (A) and CRND8 (B) lamina II dorsal horn (DH) spinal neurons: the amplitude (E), frequency (F), rise time (G), and decay time (H) of sEPSCs were not significantly different in non-Tg (*n* = 7) and CRND8 (*n* = 10) neurons. AMPA receptor-mediated mEPSCs were recorded from the same non-Tg (C) and CRND8 (D) neurons in the presence of TTX (0.1μM). In these conditions, mEPSC frequency (F) in CRND8 neurons was significantly enhanced with respect to non-Tg (*p = 0.08, *t* = 1,437 df = 13.76), whereas the amplitude (E), rise time (G), and decay time (H) of mEPSCs were not significantly different in CRND8 and non-Tg neurons. E-H) Data are represented as mean ± SEM in bar graphs showing individual values. *p < 0.05, one-tailed *t* test with Welch’s correction, CRND8 versus non-Tg.

Spinal cord slices were perfused with ACSF containing D-AP5 (50μM, an NMDA glutamate receptor antagonist), bicuculline (5μM, a GABA receptor antagonist) and strychnine (10μM, a glycine receptor blocker) in order to isolate spontaneous excitatory post synaptic currents (sEPSCs) mediated by activation of AMPA receptors for glutamate. AMPA receptor-mediated sEPSCs were recorded under voltage clamp at a holding potential (HP) of -70 mV from lamina II DH neurons visually identified in non-Tg (Fig. 10A) and CRND8 (Fig. 10B) mouse spinal cord slices. We did not observe any significant difference between non-Tg (*n* = 7) and CRND8 neurons (*n* = 10) concerning sEPSC amplitude (19.96±1.1 versus 21.09±1.3 pA, mean ±SEM, *p* = 0.26; Fig. 10E), frequency (0.89±0.26 versus 1.13±0.38 Hz, mean ±SEM, *p* = 0.30, Fig. 10F), rise time (1.10±0.16, versus 1.04±0.11 ms, *p* = 0.38, Fig. 10 G) and decay time (1.26±0.19 versus 1.41±0.09 ms, *p* = 0.24; Fig. 10 H). On the same neurons, we next applied tetrodotoxin (TTX, 0.1μM) to block synaptic activity and record AMPA receptor-mediated miniature EPSCs (mEPSCs) due to intrinsic release probability from pre-synaptic terminals (Fig. 10 C, D). In these conditions, mEPSC frequency in CRND8 neurons was significantly increased with respect to non-Tg (0.33±0.08 versus 0.58±0.15 Hz, non-Tg versus CRND8, *p* = 0.08, *t* = 1,437 df = 13.76, Fig. 10F), indicating an increased probability of glutamate release from presynaptic fibers onto lamina II DH neurons. We did not observe any significant difference between non-Tg (*n* = 7) and CRND8 neurons (*n* = 10) concerning mEPSC amplitude (18.41±0.78, versus 19.11±0.93 pA, non-Tg versus CRND8, *p* = 0.28, Fig. 10E), rise time (1.09±0.15 versus 1.03±0.12 ms, *p* = 0.38, Fig. 10G) and decay time (1.40±0.16, versus 1.44±0.04 ms, *p* = 0.42, Fig. 10H), suggesting that the number and the kinetic properties of post-synaptic AMPA receptors were not modified. These results indicated that glutamate-mediated transmission onto spinal DH neurons from CRND8 mice was enhanced at a pre-synaptic level.

## DISCUSSION

The nociceptive behavior of different transgenic animal models of AD has been assessed with conflicting results. CRND8 (double-mutant AβPP K670 N/M671 L and V717F) male mice exhibit a reduced sensitivity to both acute noxious heat stimuli and peripheral inflammatory pain starting from 3 months of age [9], when Aβ deposits are present in the brain [11]. TASTPM (double-mutant AβPPswe×3 PS1.M146 V) mice, both male and female, show a reduced sensitivity to acute noxious heat stimuli but respond normally to peripheral inflammatory pain [8]. Finally, 3xTg-AD (PS1M146 V, AβPPswe, tauP301 L transgenes) mice, both male and female, show a preserved response to thermal pain from asymptomatic to advanced stages of the disease, with females exhibiting an increased sensory-discriminative pain sensitivity than their non-Tg counterpart [10].

In the present manuscript, we used 18-week-old female CRND8 mice. At this age, CRND8 mice exhibit frank Aβ pathology and cognitive deficits [11], thus any changes in pain susceptibility could depend on both altered perception and altered encoding of noxious stimuli. Since CRND8 females and males produce equal amounts of Aβ [11], pain responses in the two sexes are not expected to depend on a different Aβ burden. We decided to use female mice because, among rodents, females are more sensitive than males to painful stimuli and less susceptible to stress-induced analgesia [12].

We demonstrated the presence of intracellular AβPP/Aβ in the spinal cord and in the DRG of CRND8 mice, which could represent a very early stage of AD-like pathology at these sites [27]. Aβ_40_ cellular immunolabeling confirmed the presence of AβPP-derived Aβ peptides both in the DH and in the DRG of CRND8 animals. The DH of non-Tg mice also exhibited some cellular labeling for Aβ_40_, probably due to the cross-reactivity between mouse and human Aβ associated to a substantial endogenous tone of Aβ_40_.

Despite the presence of intracellular AβPP/Aβ in the ventral horn, the gross locomotor function of CRND8 mice was not impaired. We did not carry out tests to assess subtle motor deficits. The expression of AβPP/Aβ in DRG peptidergic nociceptive neurons suggested that mice response to noxious thermal stimuli could be affected. Accordingly, female CRND8 mice showed delayed withdrawal responses to heat stimuli as female TASPTM mice do [8]. In TASPTM mice, the increased threshold to thermal pain was attributed to the increased endogenous tone of enkephalins in the DH. One difference with our study is that CRND8 mice did not express more enkephalins than non-Tg littermates and even the basal content of POMC, the precursor of β-endorphin, did not differ between CRND8 and non-Tg mice.

Interestingly, the frequency of AMPA-mediated mEPSP recorded from the superficial DH nociceptive neurons of both CRND8 and non-Tg mice suggested an increased spontaneous glutamate release from presynaptic afferents in CRND8 mice. This finding is in agreement with what has been observed in other neuronal systems, including the demonstrations that human AD brain-derived intracellular Aβ oligomers increase the probability of spontaneous glutamate release at presynaptic sites [28] and that increasing extracellular Aβ enhances synaptic vesicle release [29].

Because of the increased spontaneous release, the ambient concentration of glutamate is expected to increase and favor the excitability of DH nociceptive neurons in CRND8 mice. However, it has been reported that in the long term, an excessive spontaneous release could lead to the depletion of neurotransmitter vesicles [30], thus compromising the synaptic activity of primary afferents in CRND8 mice. Although speculative and not proven at moment, the impaired synaptic activity of primary afferents could be responsible for the reduced sensitivity of CRND8 mice to heat stimuli. We must point out that electrophysiological recordings were performed on spinal cord slices obtained from 11-week-old rather than 18-week-old mice, because it was easier to obtain intact slices from younger mice. Since 11-week-old CRND8 mice show a remarkable Aβ production and a pronounced cognitive impairment in the absence of plaques [11], which are missing also in the spinal cord of 18-week-old mice, it is unlikely that the younger age of the animals influenced the results of the electrophysiological recordings.

Previous work has shown that CRND8 male mice have a reduced sensitivity to thermal pain, as we found in CRND8 females, and recover faster than wild-type littermates from the peripheral inflammatory pain induced by intraplantar injection of complete Freund’s adjuvant (CFA) [9]. Instead, we have found that female CRND8 mice exhibited an increased sensibility to inflammatory pain in the late phase of the formalin test. Apart from the possible differences due to the different sex of the mice used in that and in this study, the results from the two tests are not easily comparable. In that study, the thermal and mechanical hyposensitivity of CRND8 mice was not found until 24 h after CFA injections [9], whereas we assessed susceptibility to formalin by measuring spontaneous pain behavior within 60 min of injection. We found the existence of an increased sensitivity to nociception in CRND8 mice with respect to non-Tg littermates, but only in the late phase of the formalin test, which reflects the sensitization of DH neurons to the noxious stimulus [21]. This finding is consistent with our hypothesis that the basal synaptic activity of primary afferents in CRND8 mice could be impaired and, therefore, DH neurons would respond to a strong formalin-induced peripheral stimulation able to overcome the deficit under a local facilitating condition, as discussed below in more details.

We failed to demonstrate a biochemical correlate of formalin-induced nociceptive stimulation (either p-ERK or Fos) in the spinal cord of CRND8 mice. Both p-ERK and Fos have a transient expression following formalin injection [22, 31, 32], with p-ERK expression being much faster [33]. Most likely, the tissue analysis that followed the studies of nociceptive behavior was late compared to the activation times of ERK, which we were not able to catch in non-Tg mice either. Instead, we were able to detect the induction of Fos in non-Tg mice likely because this occurred in a different time frame than in CRND8 mice.

Remarkably, we found that formalin increased the expression of POMC, the precursor of β-endorphin, in non-Tg mice but not in CRND8 mice, suggesting that this failed increase could be the factor facilitating the hyperalgesia that CRND8 mice exhibited in the late phase of the formalin test. We also found that basal levels of POMC did not differ between non-Tg and CRND8 mice, in agreement with the knowledge that endogenous analgesic opioids are not tonically active but are triggered by stressors [34]. Since POMC/β-endorphin are involved in spinal pain processing also through descending projections [25], it is possible that the failed increase of POMC reflects the presence of Aβ pathology in the hypothalamus of CRND8 mice. Of note, β-endorphin levels are found to be reduced in the CSF of AD patients and their decline correlates with dementia stage [35].

Overall, consistently with previous studies carried out in Tg mouse models of AD [8, 9], we found that CRND8 mice, at an age when the disease is established, had a reduced sensitivity to acute noxious heat stimuli. Specifically, we did not measure the stimulus intensity threshold, rather the tolerance to a suprathreshold thermal stimulus. The thermal tolerance was found to be increased in CRND8 mice, resembling the evidence that AD patients, when compared to sex- and age-matched cognitively intact controls, are less sensitive to both mild and moderate pain [36]. Moreover, to our knowledge, ours is the first evidence of an AD mouse model showing hyperalgesia to tonic inflammatory pain that requires spinal processing. Interestingly, the evidence is consistent with the observation that pain responses that do not rely on the ability of patients to communicate are generally increased in demented patients [37], pointing to changes in pain processes accompanying AD pathology.

A limit of this work, as of others, lies in the difficulty of comparing the pathology of the transgenic animal to the human one, outside the brain. AβPP has been detected in neurons of human DRG [38], but we have no more detailed information. In addition, pathological changes in the spinal cord of AD patients have not been comprehensively studied [39], with scattered evidence of both Aβ deposition [40] and abnormal tau phosphorylation [41], and with a recent MRI study showing that the spinal cord is atrophic in established AD patients [42].

The suggestion that emerges from our study is that understanding the precise nature of pain processing alterations in CRND8 mice could be useful to develop improved diagnostic and therapeutic approaches for pain in individuals with AD.

## Data Availability

The data supporting the findings of this study are available on request from the corresponding author. Data sharing is not applicable to this article as no datasets were generated or analyzed during this study.
